# Retraction: Changes in Innate and Permissive Immune Responses after HBV Transgenic Mouse Vaccination and Long-Term-siRNA Treatment

**DOI:** 10.1371/journal.pone.0261693

**Published:** 2021-12-16

**Authors:** The PLOS ONE Editors

Following the publication of this article [1], concerns were raised regarding results presented in Figs 1, 2, 4, and 6. Specifically:

- Similarities were noted between lanes within the following panels:
  ○ Fig 1D: p5.1-C2, and 5S rRNA panel.
  ○ Fig 2D, left panels: the 0.7kB results in lanes 5 and 6.
  ○ Fig 2D, right panels: HBV-C product, HBV-S product, β-actin.
- There appear to be similarities between select regions of blot background areas in the left Fig 2D panels and the Fig 3C TLR-3, TLR-8, and GAPDH panels.
- The following FACS results appear more similar than would be expected from independent samples:
  ○ Fig 4A, the CD86 7 month siRNAs panel and the CD80 9 month siRNAs panel.
  ○ Fig 4A, the CD80 5 month siRNAs panel and the CD80 7 month siRNAs panel.
  ○ Fig 6B, the Balb/c+Vac. CD3-PerCP panel and the Fig 6B TM+Vac.+CpGs CD3-PerCP panel.
  ○ Fig 6B, the upper left quadrant of the TM+Vac. CD4-FITC panel and the upper left quadrant of the TM+Vac.+CpGs CD4-FITC panel.
  ○ Fig 6B the bottom left and right quadrants of the Balb/c+Vac.+CpGs panel and the bottom left and right quadrants of the TM+Vac.+CpGs CD4-FITC panel.
- Clusters within the following FACS results appear similar:
  ○ Fig 4A, the CD80 5 month siRNAs+vac. panel.
  ○ Fig 4A, the CD80 7 month siRNAs+vac. panel.
  ○ Fig 4A, the CD80 9 month siRNAs+vac. panel.

The corresponding author disagrees with the concerns raised by the journal and stands by the published results. Furthermore, the corresponding author stated that the original data underlying the published results are no longer available.

The above issues call into question the reliability of the reported results. Therefore, the *PLOS ONE* Editors retract this article.

GLR, GYH, HZ, HHM, MCX, HBZ, WYZ, YGZ, DYS, WKH, and JL did not agree with the retraction. YF either did not respond directly or could not be reached. GLR, HZ, MCX, HBZ, WYZ, and YGZ stand by the published results.
